# Word Distance Affects Subjective Temporal Distance

**DOI:** 10.3389/fpsyg.2021.785303

**Published:** 2021-12-15

**Authors:** Cheng Wang, Yu Liu, Jun Wang

**Affiliations:** Department of Psychology, Zhejiang Normal University, Jinhua, China

**Keywords:** word distance, time perception, temporal bisection task, *lexical kappa effect*, internal clock

## Abstract

The kappa effect is a well-reported phenomenon in which spatial distance between discrete stimuli affects the perception of temporal distance demarcated by the corresponding stimuli. Here, we report a new phenomenon that we propose to designate as the *lexical kappa effect* in which word distance, a non-magnitude relationship of discrete stimuli that exists in the lexical space of the mental lexicon, affects the perception of temporal distance. A temporal bisection task was used to assess the subjective perception of the time interval demarcated by two successively presented words. Word distance was manipulated by varying the semantic (Experiment 1) or phonological (Experiment 2) similarity between the two words. Results showed that the temporal distance between the two words was perceived to be shorter when the corresponding two words were lexically closer. We explain this effect within the internal clock framework by assuming faster detection of the word that terminated timing when it is preceded by a semantically or phonologically similar word.

## Introduction

The perception of time is a universal, continuous experience, which is central to virtually all behaviors (Paton and Buonomano, 2018). Literatures show that the subjective duration of a given stimulus can be influenced by many non-temporal physical factors, such as spatial size, weight, moving velocity, luminance, and loudness of the stimulus (see Winter et al., 2015; Matthews and Meck, 2016; for reviews). Among these physical factors, one of particular relevance is spatial distance, whose influence on time perception has been demonstrated in the well-known “kappa effect”. Generally speaking, the kappa effect is such that when two brief flashes are successively presented at different locations, increasing the spatial distance between the two locations results in longer perceived temporal distance between the two flashes (Cohen et al., 1953; Price-Williams, 1954). This effect has also been demonstrated in auditory (Grondin and Plourde, 2007; Sarrazin et al., 2007) and tactile (Suto, 1952; Grondin et al., 2011) modalities. An influential account of this effect is the theory of magnitude (ATOM, Walsh, 2003; Bueti and Walsh, 2009; but see Jones and Huang, 1982 for another account) which assumes that time, space, number, and other dimensions are processed using a common magnitude system. The magnitude of space can bias the perceived magnitude of time because the perceptions of both dimensions are represented by the same magnitude system. The kappa effect shows the dependence of perceived time on spatial distance, which is a magnitude in our physical world. Here, we report a new phenomenon that time perception is affected by word distance in the mental lexicon, which is a non-magnitude distance that exists in our psychological world.

Mental lexicon is a mental store of words that contains information regarding words’ meaning, pronunciation, spelling, and syntactic characteristics (Elman, 2004; Castro and Stella, 2019). It plays a central role in language comprehension and production (Aitchison, 2012; Wang and Zhang, 2021). Models of mental lexicon assume that the information stored in it is structured as a large-scale network consisting of *nodes* (or vertices) that correspond to words and *connections* (or edges) that represent the relations between two words (Collins and Loftus, 1975; Steyvers and Tenenbaum, 2005; De Deyne et al., 2017). The connection between words is a multiplexed system, with different layers of relations based on semantic similarity (taxonomic or associative relation, e.g., *dog*—*cat*, *dog—hunt*) or phonological similarity (word-form overlapping, e.g., *dog—log*) (Stella et al., 2018; Castro and Stella, 2019). Networks based on semantic (Steyvers and Tenenbaum, 2005; De Deyne et al., 2017) or phonological (Coleman, 1998; Carlson et al., 2014) connections, or both connections simultaneously (Stella et al., 2018; Castro and Stella, 2019) have been successfully used to model various language phenomena.

The activation spreading model (Collins and Loftus, 1975) of mental lexicon assumes that activation spreads in the lexical network from one node to others, and the spreading activation dissipates with distance. The distance between the nodes (i.e., word distance) is determined by the degree of semantic or phonological similarity between the words, such that word distance decreases as word similarity increases (Collins and Loftus, 1975; den Heyer and Briand, 1986; Prabhakaran et al., 2014). For example, the node representing the word *dog* will be closer to the node representing the word *cat* or *log* than to the node representing the word *cup*. The model predicts that when a node is activated, close nodes will receive larger activation than distant ones, thereby facilitating the processing of the words represented by the closer nodes. Accordingly, a number of studies reported that presenting a prime word (e.g., *dog*) facilitated comprehending a target word (e.g., *cat* or *log*) that had semantic or phonological similarities to the prime word (Hillinger, 1980; Rouibah et al., 1999; Zhou and Marslen-Wilson, 2000). These findings of semantic and phonological priming effects demonstrated that the effects of word distance could be obtained by manipulating semantic and phonological similarities.

The current study aims to investigate whether the word distance in the mental lexicon affects time perception. Two words were successively presented briefly, and participants were asked to judge the temporal distance between the two words as long or short. Word distance between the two words were varied such that the two words were either close or distant, via manipulating semantic (Experiment 1) or phonological (Experiment 2) similarity (Hillinger, 1980; Rouibah et al., 1999; Zhou and Marslen-Wilson, 2000). We used the classic temporal bisection task (Penney et al., 2008; Matthews and Meck, 2016; Li et al., 2021) to examine the subjective temporal distance. In this task, participants are first presented with multiple “short” and “long” anchor time intervals, followed by a test phase in which they judge a set of intermediate intervals (aka, the probe intervals) as being closer to the “short” or “long” anchor intervals. Behavior performances were then fit to a psychometric curve to estimate the point of subjective equality (PSE). Larger PSEs indicate shorter perceived time (see “Materials and Methods” section for details). If the “kappa effect” exists also in the lexical space of the mental lexicon, we expect to observe that the temporal distance would be perceived to be shorter (i.e., larger PSEs) when it is flanked by a pair of close words than when flanked by a pair of distant words.

## Materials and Methods

### Participants

We conducted an *a priori* power analysis using G*Power (Faul et al., 2007) to determine the necessary sample size. This analysis (two dependent means, *cohen*’*s d* = 0.5,*alpha* = 0.05,*power* = 0.8, two tails) gave a minimum sample size of 34 participants. Experiment 1 (15 males, mean age 22.50 ± 3.28 years) and Experiment 2 (12 males, mean age 21.65 ± 2.71 years) each recruited 34 participants. For each experiment, we collected data until 34 participants met our inclusion criteria. Three and four participants were replaced in Experiments 1 and 2, respectively, following the exclusion criteria described in the “Data Analysis” section. All participants were native speakers of Mandarin Chinese, had normal hearing and normal or corrected-to-normal vision. All participants gave written informed consent before the experiment and received monetary compensation. This study was approved by the Research Ethics Committee of Zhejiang Normal University.

### Material

We used word similarity to manipulate word distance (Close vs. Distant) in the lexical network of mental lexicon (Collins and Loftus, 1975; den Heyer and Briand, 1986; Prabhakaran et al., 2014). Two conditions of word pairs were used. In the Close condition the two words in a pair had semantic (Experiment 1) or phonological (Experiment 2) similarity, while in the Distant condition the two words had no similarity. All words were Chinese two-character words. First, 25 words were selected as primes. Then, each prime word was paired with two target words. One of them had similarity with the prime (the Close condition), and the other had no similarity with the prime (the Distant condition). In Experiment 1, the pair of prime and target words in the Close condition had a semantic similarity (taxonomically related, i.e., belonging to the same category) but no other similarity, for example, 樱桃(“cherry”,/ying1tao2/)—草莓(“strawberry”,/cao3mei2/). In Experiment 2, the pair of prime and target words in the close condition had a phonological similarity (their first characters had the same pronunciation) but no other similarity, e.g., 梳子(“comb”,/shu1zi5/)—书展(“book fair”,/shu1zhan3/). Taxonomic relation (Ouyang et al., 2019; Rose et al., 2019) and phonological overlapping (Zhou and Marslen-Wilson, 2000; Zhu et al., 2015) were abundantly used to define semantic and phonological similarities, respectively, in language studies. In both experiments, the pair of prime and target words had no similarity, e.g., 樱桃—老虎(“tiger”,/lao3hu3/), and 梳子—海豚(“dolphin”,/hai3tun2/). The target words were matched between the two conditions on word frequency (|*t|* s < 1.398, *p*s > 0.175) and number of strokes (| *t|* s < 1.417, *p*s > 0.169). Note that we did not employ a priming paradigm, and naming the two words in a pair as prime and target is just for the convenience of description.

### Procedure

**FIGURE 1:**
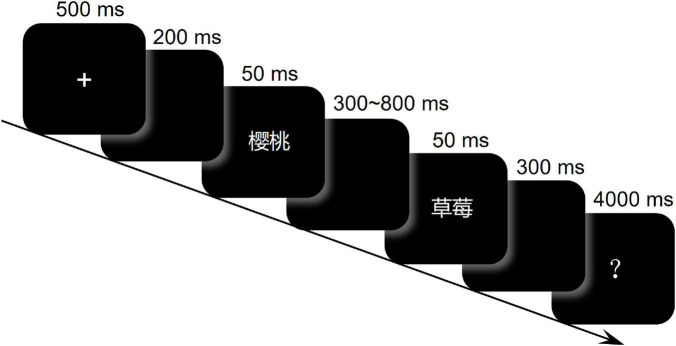
Schematic of procedure.

We used the temporal bisection task to examine the subjective temporal distance. The temporal distance was defined as the time interval demarcated by two successively presented Chinese two-character words (font: Microsoft YaHei; RGB: 192 192 192; visual angle: 1.3° × 1.3°) (see Figure 1). The two words each appeared 50 ms, and the time interval separating them varied from 300 to 800 ms. The short and long anchor intervals were 300 and 800 ms, and the probe intervals were linearly spaced from 300 to 800 ms in steps of 100 ms (i.e., 300, 400, 500, 600, 700, and 800 ms). All stimuli were presented against a black background, on a 24-inch LED monitor (resolution: 1,920 × 1,080; refresh rate: 100 Hz) at a viewing distance of 60 cm.

The temporal bisection task was programmed in E-prime (Psychology Software Tools, Sharpsburg, PA, United States). This task included a training phase followed by a testing phase. In the training phase, after clear instruction about whether the subsequently presented anchor intervals would be “long” or “short” (e.g., “Next are long intervals”), each of the anchor intervals (as demarcated by successive brief presentations of “米米”) was successively presented three times. To confirm that participants could differentiate the two anchor intervals, each anchor interval was then presented three times with a randomized order, and participants were asked to categorize them as “long” or “short.” Participants passed the training phase only after making 100% correct responses. Those who made errors would receive another round of training phase. In the testing phase, each trial (see Figure 1) began with a fixation (+) at the center of the screen for 500 ms, followed by a blank screen for 200 ms. After that, two words were successively presented, each for 50 ms. The interval separating the two words was selected from the probe intervals (i.e., 300, 400, 500, 600, 700, and 800 ms). Then, after a blank of 300 ms, a question mark appeared at the center of the screen for 4,000 ms. Participants were asked to judge, upon seeing the question mark, whether the time interval between the two words as being closer to the “long” or “short” anchor interval. The next trial began 500 ms after participant making response. Each of the six probe intervals was presented with each of the 26 pairs of words for each condition (Close and Distant), resulting 300 trials. Trial order was randomized across participants. These trials were administered in two blocks that were separated by a break. Each block consisted of 150 trials and 4 warm-up trials. Each experiment lasted approximately 25 min.

### Data Analysis

**FIGURE 2:**
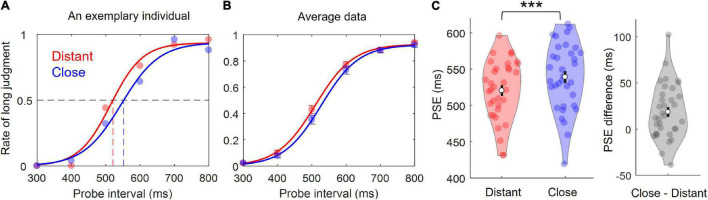
Results of Experiment 1. **(A)** Individual’s rates of “long” judgment as function of probe intervals (300–800 ms) in the Distant and Close conditions. A logistic curve was fit to each individual’s distribution of responses to derive an estimation of the point of subjective equality (PSE). **(B)** Logistic curves fit to grand mean rates of “long” judgment. Dots represents mean, and error bars indicate ± SEM. **(C)** Violin plots for the PSE. Colored dots represent individual data points. White dots represent averages. Error bars indicate ±SEM. ***p < 0.001.

**FIGURE 3:**
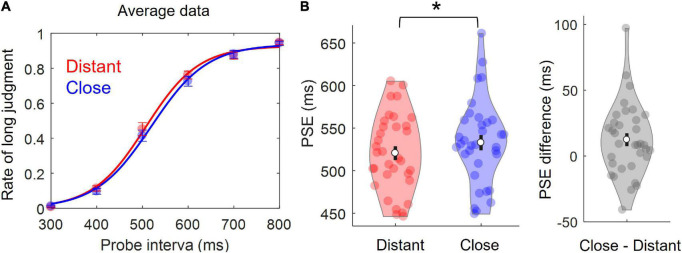
Results of Experiment 2. **(A)** Logistic curves fit to grand mean rates of “long” judgment. Dots represents mean, and error bars indicate ±SEM. **(B)** Violin plots for the PSE. Colored dots represent individual data points. White dots represent averages. Error bars indicate ±SEM. *p < 0.05.

For each participant, the rate of “long” judgment was calculated for each probe interval for each condition. The observed distribution of responses was fit to a psychometric logistic function (Treutwein and Strasburger, 1999). PSE was then calculated based on the 50% point in the obtained logistic curve. Larger PSEs indicate shorter perceived time. Figure 2A shows this process for an exemplary individual. Individual rates at each probe interval were averaged across participants, and submitted to the same fitting procedure to produce the grand average fitted curve, as shown in Figures 2B, 3B. If any data of an individual were unable to fit with a logistic function (*R*^2^ < 0.85), all data from this individual were discarded.

## Results

### Experiment 1

In the exemplary individual shown in Figure 2A, the distribution of response was clearly more left-shifted in the Distant than Close conditions, resulting in a smaller PSE in the Distant condition. Individual and mean PSEs for each condition and condition differences are shown by the violin plots in Figure 2C. Importantly, paired *t*-tests revealed that PSE was significantly smaller in the Distant condition (*M* = 520*ms*, *sd* = 41*ms*) than in the Close condition (*M* = 539*ms*, *sd* = 46*ms*), *t*_(33)_ = −3.66,*p* < 0.001, *Cohen*’*s d* = 0.628. These results indicated that the temporal distance between two successively presented words was judged to be shorter in the Close than Distant conditions, and that the semantic similarity between the two words compressed time perception.

### Experiment 2

As shown in the average data in Figure 3A, the distribution of responses was clearly more left-shifted in the Distant than Close conditions, resulting in a smaller PSE in the Distant condition. Individual and mean PSEs for each condition and condition differences are shown by the violin plots in Figure 3B. Importantly, paired *t*-tests revealed that PSE was significantly smaller in the Distant condition (*M* = 521*ms*, *sd* = 45*ms*) than in the Close condition (*M* = 533*ms*, *sd* = 50*ms*), *t*_(33)_ = −2.64,*p* = 0.013, *Cohen*’*s d* = 0.453. These results indicated that the temporal distance between two successively presented words was judged to be shorter in the Close than Distant conditions, and that the phonological similarity between the two words compressed time perception.

To assess whether the effects of word distance on temporal distance were different across the two experiments, we conducted a 2 × 2 mixed-design ANOVA with type of word distance (semantic vs. phonological) as a between-subject factor and word distance (close vs. distant) as a within-subject factor. The results showed that only the main effect word distance was significant, $F=20.08,p<0.001,\eta_{p}^{2}=0.233$. The main effect of type ($\text{F = 0.09},p=0.772,\eta_{p}^{2}=0.001$) and the interaction between type and word distance ($F=0.81,p=0.371,\eta_{p}^{2}=0.012$) were both not significant. The absence of interaction indicated that the effects of word distance on temporal distance were comparable across the two types of word distance.

## Discussion

To investigate whether the “kappa effect” originally observed in the physical space can extend to the lexical space of the mental lexicon, two experiments using the temporal bisection task were conducted to examine whether the word distance affects time perception. We observed that the temporal distance between two successively presented words was perceived to be shorter when the corresponding two words were semantically or phonologically close in the mental lexicon, compared to when they were distant. These results indicate that two successively presented words appear to be temporally closer when they are lexically closer. We hereby propose to designate this new phenomenon as the *lexical kappa effect*, and explain this effect within the framework of internal clock models (Gibbon et al., 1984; Wearden, 2004; Treisman, 2013).

The original kappa effect showed that the temporal distance flanked by discrete stimuli was perceived to be shorter when the flanking stimuli were more closely spaced (Cohen et al., 1953; Price-Williams, 1954; Sarrazin et al., 2007). Likewise, the lexical kappa effect observed in the present study revealed that the temporal distance between the two successively presented words was perceived to be shorter when the word distance between the two flanking words was smaller (i.e., semantically or phonologically similar). Both effects show that a non-temporal distance between two stimuli flanking a temporal distance can bias the perceived duration of that temporal distance in the same direction as that of the non-temporal distance. But the non-temporal distance involved in the two effects are vastly different. In the original kappa effect the non-temporal distance is spatial distance, which is a tangible, daily-used magnitude in the physical space (Cohen et al., 1953; Price-Williams, 1954; Sarrazin et al., 2007), while in the lexical kappa effect the non-temporal distance is word distance, which is a non-magnitude, between-word relation in the lexical space of the mental lexicon.

The kappa effect is a special variant of a broader, general bias that time interval flanked by discrete stimuli is perceived to be shorter when difference between the stimuli is smaller (e.g., Cohen et al., 1953; Alards-Tomalin et al., 2014; Leboe-McGowan et al., 2021). In almost all previous studies examining this bias, the difference between the flanking stimuli were manipulated on magnitude across a variety of dimensions, including space (Cohen et al., 1953; Price-Williams, 1954; Sarrazin et al., 2007), pitch height (Shigeno, 1993; Henry and McAuley, 2009; Marty et al., 2021), object size (Xuan et al., 2007; Matthews, 2011), digital number (Alards-Tomalin et al., 2014), and color saturation (Alards-Tomalin et al., 2014). A general framework of ATOM (Walsh, 2003; Bueti and Walsh, 2009) was used to overall explain these magnitude-based biases (Alards-Tomalin et al., 2014). ATOM proposes that time, space, number, and other dimensions are processed as a common magnitude in the intraparietal sulcus of the brain. According to ATOM, the magnitude of one dimension of stimuli (e.g., spatial distance) interferes with the perceived magnitude of another (e.g., temporal distance) because the magnitude of both dimensions is represented in the same brain module.

Unlike the above studies, the lexical kappa effect is a time perception bias that was rooted in a non-magnitude dimension, i.e., the lexical space of the mental lexicon. We manipulated the difference of flanking stimuli on word distance, which was essentially semantic or phonological relatedness between words and cannot be characterized as a magnitude. Thus, our results cannot be explained by the ATOM theory, as it relies on magnitude difference of stimuli. A recent study (Leboe-McGowan et al., 2021) also excluded ATOM from explaining distorting effects from non-magnitude dimensions. In this study, stimuli flanking a time interval were two letters that either shared the same identity or not (Experiment 1), a color word and a colored rectangle that either described the same color or not (Experiment 2), or a sentence stem and a terminal word that fit either well or poorly (Experiment 3). In all cases, Leboe-McGowan et al. (2021) found that letter or conceptual overlap (i.e., smaller difference) between flanking stimuli compressed time perception. They argued that ATOM was not applicable for explaining their findings because letter and conceptual overlap were not magnitude and ATOM required shared representation of magnitude.

Instead, we attempt to explain the lexical kappa effect within the framework of internal clock models (Gibbon et al., 1984; Wearden, 2004; Treisman, 2013). According to these models, the subjective perception of time is computed by an internal clock mechanism that consists of a pacemaker, a switch and an accumulator. The pacemaker emits at a given rate pulses that flows into the accumulator, and the switch controls the gate of the pulse flow. At the onset of timing, the switch closes, allowing pulses to flow into the accumulator; at the offset of timing, the switch opens and stops the flow. The number of pulses accumulated during the event(s) being timed represents the duration of the time. The more pulses are accumulated, the longer the duration is perceived to be. In this framework, non-temporal features of stimuli can affect the latency to close or open the switch, or the pacemaker rate, thereby producing a change in its judged duration.

In the current study, the to-be-timed intervals were demarcated by two successive words. The initiating words signaled the switch to close, and the terminal words signaled the switch to open. Before participants became sensitive to the terminal words, the responses of the internal clock (i.e., latency to close switch and pacemaker rate) across the two conditions should be equivalent, because the initiating words and the display during the to-be-timed interval were identical across conditions. Thus, the effect of word distance could only be explained by different latencies to open the switch at the end of timing, which were signal by the terminal words. The terminal words had a semantic (Experiment 1) or phonological (Experiment 2) similarity with the initiating words in the Close condition, but had no similarity with the initiating words in the Distant condition. As word similarity not only facilitates word recognition (Hillinger, 1980; Rouibah et al., 1999; Zhou and Marslen-Wilson, 2000) but also increases perceptual sensitivity of words (Rhodes et al., 1993; Pastore et al., 2003), we can infer that the terminal words were detected faster in the Close condition than in the Distant condition, which would lead to relatively earlier latencies to open the switch and hence less pulses transferred into the accumulator in the Close condition. If so, it would lead participants to judge time intervals flanked by similar words as shorter. Some other studies adopted a similar explanation to explain their observations of shortening effect of flanking stimuli (Grondin et al., 1996; Matthews, 2011; Leboe-McGowan et al., 2021). For example, Grondin et al. (1996) observed that time intervals were judged as shorter when they were flanked by a visual signal followed by an auditory signal than when flanked by two successive visual signals, and they explained this effect by assuming that the offset of the interval was detected faster when it was marked by an auditory signal.

The faster processing of the terminal words when preceded by a semantically or phonologically similar words can be explained under the spreading activation model of the mental lexicon (Collins and Loftus, 1975). In the lexical network, word distance is shorter for similar words than for dissimilar words. When the initiating words were activated, activation spread along the network and dissipated with distance. Compared to terminal words in the Distance condition, terminal words in the Close condition were closer to the initiating words, and therefore received larger spreading activation. This could have facilitated the detection of the terminal words, and resulted in earlier latencies to open the switch (i.e., end timing) of the internal clock in the Close condition. Although the network model is prevalent, alternative models had also been proposed to explain the semantic or phonological priming effects (e.g., Masson, 1995; McRae et al., 1997; McNamara, 2005). For example, the distributed memory model (Masson, 1995) assumes that a word is represented by a specific pattern of activation of a collection of processing units, and the semantic priming effect arise from similar patterns of activation that represent the prime and target words. These alternative models can also explain faster detection of the terminal words in the Close condition, and thus do not contradict the interpretation for the observed effect of word similarity on time perception within the framework of internal clock.

In conclusion, the perception of a temporal distance can be distorted by word distance in the lexical space, in a manner that smaller distance between two words shortens the perceived temporal distance demarcated by the two words. As word distance is not a magnitude property of stimulus, this bias cannot be explained by the influential ATOM theory. Within the internal clock framework, we propose to explain it by faster detection of the word that terminated timing when it is preceded by a semantically or phonologically similar word.

## Data Availability Statement

The raw data supporting the conclusions of this article will be made available by the authors, without undue reservation.

## Ethics Statement

The studies involving human participants were reviewed and approved by the Research Ethics Committee of Zhejiang Normal University. The patients/participants provided their written informed consent to participate in this study.

## Author Contributions

CW wrote the manuscript. CW and JW designed and supervised the research. YL conducted the experiments and analyzed the data. All authors contributed to the article and approved the final version for submission.

## Conflict of Interest

The authors declare that the research was conducted in the absence of any commercial or financial relationships that could be construed as a potential conflict of interest.

## Publisher’s Note

All claims expressed in this article are solely those of the authors and do not necessarily represent those of their affiliated organizations, or those of the publisher, the editors and the reviewers. Any product that may be evaluated in this article, or claim that may be made by its manufacturer, is not guaranteed or endorsed by the publisher.
